# Supplementary material for: Tissue engineered vascular grafts are resistant to the formation of dystrophic calcification
Source: Nat Commun. 2024 Mar 11;15:2187. doi: 10.1038/s41467-024-46431-4 (PMC10928115; doi:10.1038/s41467-024-46431-4)
Supplement: Supplementary file 1 — Supplementary Information [file 41467_2024_46431_MOESM1_ESM.pdf]

Supplementary Figure 1

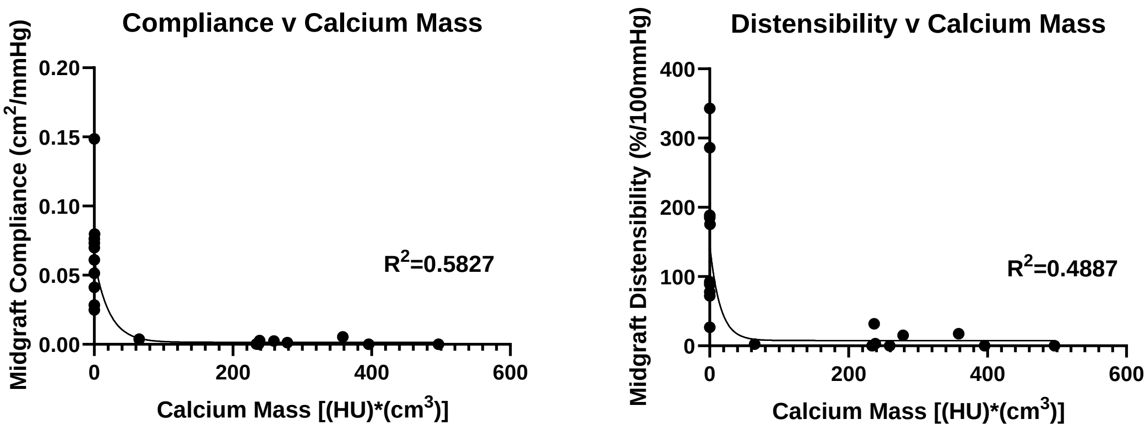

Supplementary Figure 1. Correlation of Compliance and Distensibility with Calcium Mass. Investigating the relationship between calcium mass and graft compliance and distensibility. Contrasting values of compliance and distensibility versus calcium mass revealed a highly nonlinear, not linear, relationship. An exponential decay function was found to best fit the data, with correlation coefficients of R^2=0.5827 (compliance versus calcium mass) and R^2=0.4887 (distensibility versus calcium mass). This strong exponential relationship reflects a near binary difference: marked calcification in the PTFE grafts and no calcification in the TEVGs.

Supplementary Figure 2

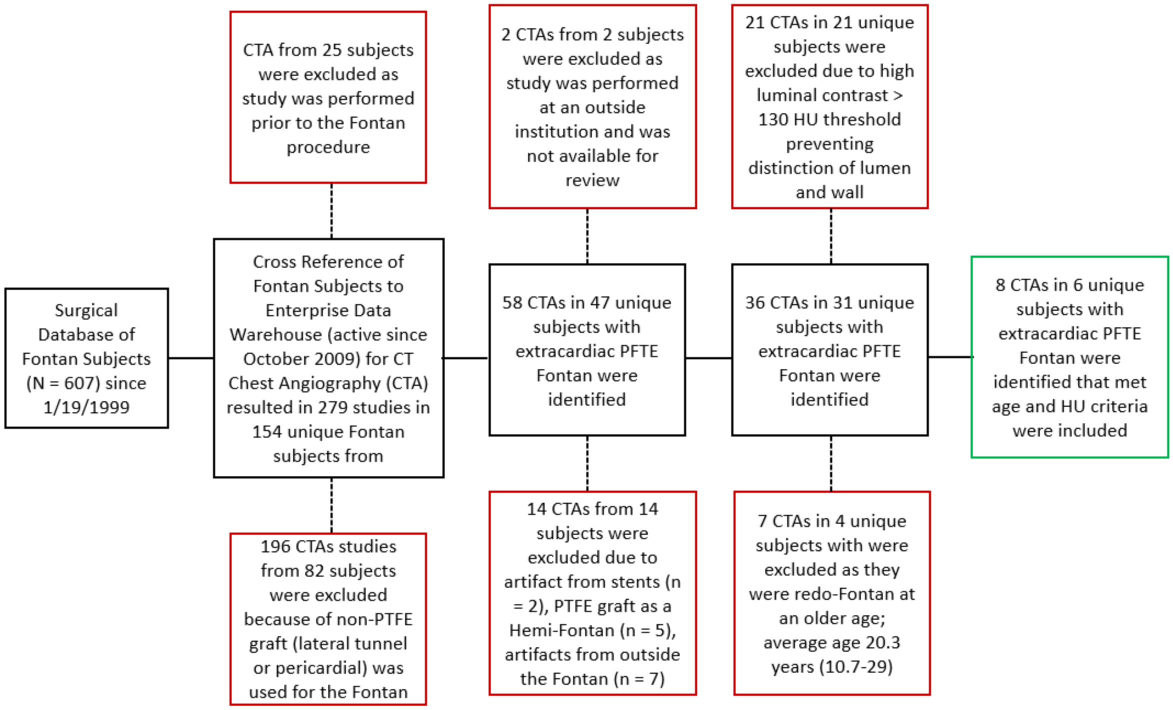

Supplementary Figure 2 Flow Chart Illustrating the Retrospective Database Search Methodology. Since the Japanese clinical trial did not include a control group, we retrospectively

obtained a control cohort through an IRB-approved (IRB14-00035) search of the Nationwide Children's Hospital surgical database. The process involved obtaining data from the NCH Heart Center Surgical Database, then cross-referencing it with CT imaging and hospital databases. Subsequently, we categorized Fontan patients who underwent CT scans with a focus on those with PTFE extracardiac Fontan conduits whose data were available for review. Further refinement excluded patients that were redo-Fontans or patients that had significant artifact due to device placement or high luminal contrast, preventing distinction of the lumen and wall. Six unique patients met the age and artifact criteria, which provided eight total scans as two of the six patients received repeat imaging.
