# Supplementary material for: Tissue engineered vascular grafts are resistant to the formation of dystrophic calcification
Source: Nat Commun. 2024 Mar 11;15:2187. doi: 10.1038/s41467-024-46431-4 (PMC10928115; doi:10.1038/s41467-024-46431-4)
Supplement: Supplementary file 3 — Reporting Summary [file 41467_2024_46431_MOESM3_ESM.pdf]

nature portfolio

Corresponding author(s): Christopher Breuer, MD

Last updated by author(s): Oct 30, 2023

Reporting Summary

Nature Portfolio wishes to improve the reproducibility of the work that we publish. This form provides structure for consistency and transparency in reporting. For further information on Nature Portfolio policies, see our Editorial Policies and the Editorial Policy Checklist.

Statistics

For all statistical analyses, confirm that the following items are present in the figure legend, table legend, main text, or Methods section.

n/a	Confirmed	
☐	☒	The exact sample size (*n*) for each experimental group/condition, given as a discrete number and unit of measurement
☐	☒	A statement on whether measurements were taken from distinct samples or whether the same sample was measured repeatedly
☐	☒	The statistical test(s) used AND whether they are one- or two-sided<br>*Only common tests should be described solely by name; describe more complex techniques in the Methods section.*
☒	☐	A description of all covariates tested
☐	☒	A description of any assumptions or corrections, such as tests of normality and adjustment for multiple comparisons
☐	☒	A full description of the statistical parameters including central tendency (e.g. means) or other basic estimates (e.g. regression coefficient) AND variation (e.g. standard deviation) or associated estimates of uncertainty (e.g. confidence intervals)
☐	☒	For null hypothesis testing, the test statistic (e.g. *F*, *t*, *r*) with confidence intervals, effect sizes, degrees of freedom and *P* value noted<br>*Give P values as exact values whenever suitable.*
☒	☐	For Bayesian analysis, information on the choice of priors and Markov chain Monte Carlo settings
☒	☐	For hierarchical and complex designs, identification of the appropriate level for tests and full reporting of outcomes
☒	☐	Estimates of effect sizes (e.g. Cohen's *d*, Pearson's *r*), indicating how they were calculated

Our web collection on statistics for biologists contains articles on many of the points above.

Software and code

Policy information about availability of computer code

Data collection	NA
Data analysis	Analyses were performed using GraphPad Prism version 9.0.0. Simulations were completed using the open-source code SimVascular, release version May 2023, available at https://simvascular.github.io.

For manuscripts utilizing custom algorithms or software that are central to the research but not yet described in published literature, software must be made available to editors and reviewers. We strongly encourage code deposition in a community repository (e.g. GitHub). See the Nature Portfolio guidelines for submitting code & software for further information.

Data

Policy information about availability of data

All manuscripts must include a data availability statement. This statement should provide the following information, where applicable:

- Accession codes, unique identifiers, or web links for publicly available datasets
- A description of any restrictions on data availability
- For clinical datasets or third party data, please ensure that the statement adheres to our policy

Data are available upon request

Research involving human participants, their data, or biological material

Policy information about studies with human participants or human data. See also policy information about sex, gender (identity/presentation), and sexual orientation and race, ethnicity and racism.

Reporting on sex and gender	Male and female patients were both included in this study.
Reporting on race, ethnicity, or other socially relevant groupings	This was a retrospective study wherein we included all patients that had an extracardiac Fontan conduit and a CT scan. Therefore, groups may include patients of different races or ethnicities. Due to the limited patient numbers and unique clinical indications for this study, further characteristics would impinge on the privacy rights of the patients in this study and have thus been excluded."
Population characteristics	This was a retrospective study wherein we included all patients that had an extracardiac Fontan conduit and a CT scan. Patients were different ages, but to the best of our abilities, we selected patients that were "age-matched" and "duration-matched." Patient ages at implantation ranged from 2.1-11.1 years for the TEVG group and 3.3-14.3 years for the PTFE group. Duration of implantation prior to CT imaging ranged from 7.4-13.2 years in the TEVG group and 0.2-16.2 in the PTFE group.
Recruitment	This was a clinical study, not a clinical trial. Due to the retrospective nature of the study, we do not have any specific recruitment details.
Ethics oversight	Ethics committee at Tokyo Women's Medical University approved the implantation of TEVGs in human subjects (TWMU IRB-198). All studies in the US were IRB approved and were overseen by the Office for Human Research Protections of Nationwide Children's Hospital (IRB STUDY00003038, IRB 14-00035)."

Note that full information on the approval of the study protocol must also be provided in the manuscript.

Field-specific reporting

Please select the one below that is the best fit for your research. If you are not sure, read the appropriate sections before making your selection.

☒ Life sciences ☐ Behavioural & social sciences ☐ Ecological, evolutionary & environmental sciences

For a reference copy of the document with all sections, see nature.com/documents/nr-reporting-summary-flat.pdf

Life sciences study design

All studies must disclose on these points even when the disclosure is negative.

Sample size	For the clinical study, the n was not prospectively established. We used a dataset of late-term clinical CT scans of the cardiothoracic cavity from the Japanese TEVG clinical trial provided by Tokyo Women's Hospital (TWMU IRB-198). The Japanese clinical trial did not incorporate a control group; thus, we retrospectively searched the electronic medical record database at our institution (Nationwide Children's Hospital; IRB14-00035) for all patients with an extracardiac PTFE Fontan conduit (GORE-TEXÒ Stretch vascular graft) who underwent non-enhanced or contrast-enhanced CT scan. We excluded studies that had visible intraluminal contrast within the Fontan conduit lumen, which could interfere with mural calcium assessment due to volume averaging. We used contrast studies with lack of visible contrast within the Fontan conduit due to the early/aortic phase of contrast enhancement since the Fontan circuit tends to fill late following an upper extremity intravenous injection of contrast. We also selected patients without a stent, pacemaker lead, or other device that caused significant artifact that could potentially interfere with attenuation measurement. Patient demographics were also obtained, and the study included both sexes. All patients with imaging studies that met the above criteria were utilized.<br>For the ovine study, the n was not prospectively established. Previous studies by our lab have evaluated the implantation of TEVGs and PTFE conduits as inferior vena cava interposition grafts (AR13-00079). All living animals that met criteria of being at least 4 years post-implantation were used in the study. In addition, all available histological samples from previously sacrificed animals were included in the study from the PTFE group, and all available histological samples from the TEVG group at the same time point were utilized as a comparison group
Data exclusions	No subjects were excluded from the ovine study. In the human clinical study, patient CT scans were excluded if 1.) the Fontan conduit was a Fontan revision, 2.) there was a device causing significant artifact in the CT scan, or 3.) They were duplicate scans of the same patient and not a distinct data point.
Replication	NA - as patients are recruited to the TEVG clinical trial, we hope to replicate this data.
Randomization	Due to the retrospective review nature of this clinical study, randomization was not possible. All available imaging studies were reviewed and included as listed in the sample size description. For the ovine studies, animals were randomized to graft material at the time of implantation (AR13-00079)
Blinding	Blinded investigation was performed for all analyses; all samples were coded so as to remove identifiers including patient age, time since implant, and graft material. This coding system was used to blind the researchers performing CT imaging, calcium scoring analysis, histological preparation and evaluation, and compliance measurement.

Reporting for specific materials, systems and methods

We require information from authors about some types of materials, experimental systems and methods used in many studies. Here, indicate whether each material, system or method listed is relevant to your study. If you are not sure if a list item applies to your research, read the appropriate section before selecting a response.

Materials & experimental systems

n/a	Involved in the study
[x]	[ ] Antibodies
[x]	[ ] Eukaryotic cell lines
[x]	[ ] Palaeontology and archaeology
[ ]	[x] Animals and other organisms
[ ]	[x] Clinical data
[ ]	[x] Dual use research of concern
[x]	[ ] Plants

Methods

n/a	Involved in the study
[x]	[ ] ChIP-seq
[x]	[ ] Flow cytometry
[x]	[ ] MRI-based neuroimaging

Animals and other research organisms

Policy information about studies involving animals; ARRIVE guidelines recommended for reporting animal research, and Sex and Gender in Research

Laboratory animals	We used ovine subjects aged 4-6 years.
Wild animals	No wild animals were used in this study
Reporting on sex	We used both male and female ovine subjects.
Field-collected samples	No field-collected samples were used in this research study
Ethics oversight	Found in Ethical statement. IACUC at Nationwide Children's Hospital (Protocol AR13-00079)

Note that full information on the approval of the study protocol must also be provided in the manuscript.

Clinical data

Policy information about clinical studies

All manuscripts should comply with the ICMJEguidelines for publication of clinical research and a completedCONSORT checklist must be included with all submissions.

Clinical trial registration	CT scans TEVG patients evaluated in this study were a part of a clinical trial at Tokyo Women's Hospital (TWMU IRB-198). US control PTFE patients were not part of a clinical trial, and were instead selected from chart review of available CTs within Nationwide Children's Hospital (Nationwide Children's Hospital; IRB14-00035).
Study protocol	Tokyo Women's TWMU IRB-198; NCH IRB14-00035
Data collection	We used a dataset of late-term clinical CT scans from the Japanese clinical trial. We retrospectively searched the electronic medical database at our institution for all patients with an extracardiac PTFE Fontan conduit that underwent CT scanning.
Outcomes	We used contrast and non-contrast CT scans that were taken prior to this study. The primary clinical and ovine outcome was defined as calcification within the graft wall, evaluated by CT using previously established imaging threshold techniques. Ovine secondary outcomes included distensibility on angiographic fluid overload stress testing.

Dual use research of concern

Policy information about dual use research of concern

Hazards

Could the accidental, deliberate or reckless misuse of agents or technologies generated in the work, or the application of information presented in the manuscript, pose a threat to:

No	Yes
[x]	[ ] Public health
[x]	[ ] National security
[x]	[ ] Crops and/or livestock
[x]	[ ] Ecosystems
[x]	[ ] Any other significant area

Experiments of concern

Does the work involve any of these experiments of concern:

No	Yes	
☒	☐	Demonstrate how to render a vaccine ineffective
☒	☐	Confer resistance to therapeutically useful antibiotics or antiviral agents
☒	☐	Enhance the virulence of a pathogen or render a nonpathogen virulent
☒	☐	Increase transmissibility of a pathogen
☒	☐	Alter the host range of a pathogen
☒	☐	Enable evasion of diagnostic/detection modalities
☒	☐	Enable the weaponization of a biological agent or toxin
☒	☐	Any other potentially harmful combination of experiments and agents

Plants

Seed stocks	*Report on the source of all seed stocks or other plant material used. If applicable, state the seed stock centre and catalogue number. If plant specimens were collected from the field, describe the collection location, date and sampling procedures.*
Novel plant genotypes	*Describe the methods by which all novel plant genotypes were produced. This includes those generated by transgenic approaches, gene editing, chemical/radiation-based mutagenesis and hybridization. For transgenic lines, describe the transformation method, the number of independent lines analyzed and the generation upon which experiments were performed. For gene-edited lines, describe the editor used, the endogenous sequence targeted for editing, the targeting guide RNA sequence (if applicable) and how the editor was applied.*
Authentication	*Describe any authentication procedures for each seed stock used or novel genotype generated. Describe any experiments used to assess the effect of a mutation and, where applicable, how potential secondary effects (e.g. second site T-DNA insertions, mosiacism, off-target gene editing) were examined.*
